# Beating cancer‐related fatigue with the Untire mobile app: Results from a waiting‐list randomized controlled trial

**DOI:** 10.1002/pon.5492

**Published:** 2020-10-11

**Authors:** Simon Sebastian Spahrkäs, Anne Looijmans, Robbert Sanderman, Mariët Hagedoorn

**Affiliations:** ^1^ Department of Health Psychology University of Groningen, University Medical Center Groningen Groningen the Netherlands; ^2^ Department of Psychology, Health & Technology University of Twente Enschede the Netherlands

**Keywords:** app, cancer, cancer survivors, fatigue, mHealth, palliative care, oncology, RCT, self‐management, quality of life, psycho‐oncology

## Abstract

**Objective:**

This waiting‐list randomized controlled trial examined the effectiveness of a self‐management mHealth app in improving fatigue and quality of life (QoL) in cancer patients and survivors.

**Methods:**

Persons with cancer‐related fatigue (CRF) were recruited across four English speaking countries, via social media, and randomized into intervention (n = 519) and control (n = 280) groups. Whereas the intervention group received immediate access to the Untire app, the control group received access only after 12‐weeks. Primary outcomes fatigue severity and interference, and secondary outcome QoL were assessed at baseline, 4, 8, and 12‐weeks. We ran generalized linear mixed models for all outcomes to determine the effects of app access (yes/no), over 12‐weeks, following the intention‐to‐treat principle.

**Results:**

Compared with the control group, the intervention group showed significantly larger improvements in fatigue severity (*d* = 0.40), fatigue interference (*d* = 0.35), and overall QoL on average (*d* = 0.32) (*P*'s < .01), but not for overall QoL in the past week (*P* = .07). Sensitivity analyses indicated that participants with medium or high app use benefited most when compared with nonusers and control participants (*P*'s ≤ .02). The intervention effect on fatigue interference was slightly stronger in younger participants (≤56 vs. >56). Effects did not depend on education and cancer status. Reliable change analyses indicated that significantly more people showed full recovery for fatigue in the intervention vs the control group (*P*'s = .02).

**Conclusions:**

The Untire app can be an effective mHealth solution for cancer patients and survivors with moderate to severe CRF.

## 1 BACKGROUND

Cancer‐related fatigue (CRF), commonly defined as a distressing, persistent, subjective sense of physical, emotional, and/or cognitive tiredness or exhaustion, is the number one side effect of cancer and its treatment, affecting cancer patients' and survivors' quality of life (QoL) profoundly.^1^, ^2^ In‐person psychosocial treatments based on cognitive behavior therapy (CBT), mindfulness‐based stress reduction (MBSR), psycho‐education, and physical exercises have been found to reduce symptoms of fatigue effectively, but are limited in reach, as one therapist can only treat one patient or group at a time.^3^ Employing eHealth interventions, or the more specific mHealth interventions (via the mobile phone, eg, apps), might help to overcome this limitation as many patients worldwide could be reached. Advantages of mHealth apps include immediate access to the app (and does not require traveling), the low‐threshold to use it (and at a moment of patients' convenience), and the focus on self‐management (without contact with a therapist, which reduces costs).^4^ Apps can be used anywhere, anytime, and for an undetermined period of time. For patients with CRF, web‐based and therapist‐guided interventions^3^, ^5^, ^6^, ^7^, ^8^, ^9^, ^10^ have shown to reduce CRF effectively. However, no mHealth interventions have been available,^3^ and empirical evidence to support the effectiveness of apps, in general, is limited.^11^

## 2 PURPOSE OF THE RESEARCH

This study examines the Untire self‐management app, which is based on clinical practice and the abovementioned evidence‐based methods for managing CRF.^5^ We study the app's effectiveness as a mHealth solution for cancer patients and survivors (ie, persons who once received the diagnosis of cancer, but stated not to have cancer at the moment) with moderate to severe levels of fatigue.

The primary aim of this RCT is:

1. To assess the effectiveness of the Untire app in reducing fatigue (as measured by the Fatigue Symptom Inventory [FSI]^12^, ^13^, ^14^) in patients and survivors after 12‐weeks of app access vs patients and survivors in a waiting‐list control group.

Secondary aims are:

1. To assess the effectiveness of the Untire app in improving QoL (as measured by 1‐item of the QoL EORTC‐QLQ‐30^15^ questionnaire, and one self‐developed item on “QoL on average”) in patients and survivors after 12‐weeks of app access vs patients and survivors in a waiting‐list control group.
2. To explore whether demographic factors and health characteristics moderate the hypothesized effect of the intervention on fatigue.

## 3 METHOD

### 3.1 Study design and setting

The Untire app study is an international waiting‐list randomized controlled trial (RCT) aimed to examine the effectiveness of the Untire app in improving levels of fatigue and QoL in patients and survivors of cancer in four countries (ie, Australia, Canada, the United Kingdom, and the United States). Details are provided in the protocol.^16^ The trial has been registered in the Trial Registry on November 29, 2017 (https://www.trialregister.nl/trial/6642).

### 3.2 Study population

The Untire app study included cancer patients and survivors of ≥18 years, who experienced fatigue at moderate/severe levels (ie, having an average composite score of ≥3^17^ on items 1‐3 of the FSI^12^, ^13^, ^14^) and owned a smartphone, tablet, or iPad (ie, iOS/Android). Exclusion criteria were a diagnosis of and receiving treatment for a severe mental disorder since these persons may need more intensive treatment than offered by the app. Also excluded were persons having a diagnosis of myalgic encephalomyelitis/chronic fatigue syndrome or fibromyalgia, since the app focusses on improving physical activity, which could be potentially harmful to these patients.^18^

### 3.3 Procedure and recruitment

From March to October 2018, we recruited potential participants online via multiple paid social media advertising campaigns on Facebook using Facebook Ads Manager, directing persons to the study landing page of the online survey tool (ie, Questback). These campaigns match to female and male Facebook users who have shown interest in topics related to cancer, fatigue, and cancer survivorship. Based on participant characteristics of those who showed interest (ie, clicked on the advertisement), comparable participants (ie, lookalike audiences) were approached in subsequent advertisement campaigns.

On the study landing page, interested persons were screened for eligibility.^16^ Based on the e‐mail address and/or name, we checked for multiple registration attempts at the eligibility screening and removed duplicate entries. Eligible persons received the information letter (online) and gave electronic informed consent. After completing a baseline questionnaire, participants were randomized into the intervention or control group. Intervention participants received a personal access code that could be used to activate the Untire app after downloading it from the app store, enabling free usage. All participants received e‐mail invitations and reminders at 4, 8, 12, and 24‐weeks to complete the questionnaires. This article focusses on the outcomes of the baseline (T0)‐T12. Once control participants completed the 12‐week questionnaire, they also received an access code that could be used to activate the app for free. All participants were allowed to seek or accept other treatments for their fatigue during the study.

### 3.4 Randomization and blinding

Participants who completed the baseline were automatically randomized 2:1 by the online survey tool into the intervention and control groups. Since intervention participants received the incentive (app access) right away, we expected more dropout in the intervention group at 12‐weeks.^19^, ^20^ The randomization ratio was chosen to balance the number of 12‐week assessments between intervention and control, and enabling a sufficient number of assessments for in‐depth analyses within the intervention group. Due to the character of the intervention, it was not possible to blind participants and researchers to the intervention or control allocation.

### 3.5 Intervention

The Untire app intervention is based on evidence‐based methods for patients with CRF in clinical practice and comprises four modules (ie, My themes, My exercises, Physical activity, and Tips), see Appendix for more information. It is hypothesized that the app addresses dysfunctional thoughts via CBT and psycho‐education (My themes), reduces stress and improves sleep via MBSR (My exercises), helps to improve physical fitness through exercise instructions (Physical Activity), and empowers via positive psychology (Tips).^3^, ^5^, ^21^, ^22^, ^23^, ^24^, ^25^, ^26^, ^27^ In addition, through Quick scans, participants get weekly insight into their levels of fatigue, burden, happiness, satisfaction, and energy leaks over time (see Appendix). Participants in the intervention group were instructed to use the app at their own pace and could freely choose modules to work on. While daily app use was recommended, participants received instructions to use the app at least once a week. We provided automatic push notifications within the first 2 weeks of app use to support participants to activate the app. In case that participants did not use the app, reminders where send.

### 3.6 Outcome assessment

#### 3.6.1 Primary outcome measure

The primary outcome was the change in fatigue severity and interference from baseline to 12‐weeks, assessed with the self‐report questionnaire FSI.^12^, ^13^, ^14^ Fatigue severity and interference were assessed by calculating the average of three severity (FSI‐items 1‐3) and seven interference items (FSI‐items 5‐11), respectively. The items were answered on an 11‐point Likert‐scale ranging from 0 (not at all fatigued/no interference) to 10 (as fatigued as I could be/extreme interference). A higher score indicates higher fatigue severity or interference.

#### 3.6.2 Secondary outcome measures

The secondary outcome was change in QoL from baseline to 12 weeks, with the 1‐item QoL EORTC‐QLQ‐30 questionnaire (*QoL during the past week* ^15^) as well as a self‐constructed item of *QoL on average* (How would you rate your overall QoL on average?), on a 7‐point Likert‐scale ranging from 0 (very poor) to 7 (excellent). A higher score indicates a better QoL.

#### 3.6.3 Moderating factors

Demographic data such as age, gender, country of residence, level of education, cancer type, and cancer status (cancer patient vs survivor) were collected as potential moderators.

#### 3.6.4 In‐app log data

Once participants activated their personal access code and gave consent to the privacy terms in the Untire app, participants were registered, and their in‐app activities were automatically stored anonymized (log data). Log data was used to determine the degree of app use, which was calculated as the number of days with ≥1 activity performed within the 12‐week intervention period.

### 3.7 Statistical analysis

#### 3.7.1 Descriptive statistics

We will present baseline characteristics (ie, demographics and health characteristics) of participants who completed the baseline fatigue assessment. Alpha was set to .05.

#### 3.7.2 Fatigue

Fatigue severity and interference were analyzed using generalized linear mixed models (GLMM) with four repeated measures (baseline, 4, 8, and 12 weeks) and two groups (intervention and control). Intention‐to‐treat (ITT) is used as the primary approach in all outcome analyses. Based on previous studies testing health‐related apps targeting behavior change,^28^, ^29^, ^30^, ^31^, ^32^, ^33^ we estimated the percentage of participants that would lose interest in the app or study assessments, and therefore would be lost to follow‐up at the 12‐weeks measurement, at 60%. Outcomes of the model run in the ITT approach and the outcomes of a model run among T12‐completers were carried out to demonstrate the robustness of the primary findings. A sensitivity analysis explored whether the degree of app use is related to the degree of change in fatigue severity or interference over time. The 33% most active participants were categorized as “high users,” the middle 33% as “medium,” the least active 33% as “low,” and intervention participants who had not downloaded the app as “nonusers.” Differences in outcomes between intervention participants who showed high, medium, or low app use, nonusers, and control participants were tested by repeating the GLMM analysis of the primary outcome, but now comparing these five groups.

#### 3.7.3 Quality of life

QoL of the past week and overall QoL on average were analyzed by repeating the same GLMM analysis of the primary outcome using the ITT approach.

#### 3.7.4 Moderators

To gain insight into whether the hypothesized intervention effect varies among different groups of participants, potential moderating factors of the intervention effect on fatigue were explored by testing a three‐way interaction (time*condition*moderator) using GLMM. Variables with ≥30 participants per category were analyzed.

#### 3.7.5 Clinical significance

To determine validly whether the degree of change in fatigue was clinically meaningful, the reliable change index (RCI) was calculated.^34^ The RCI is (pretreatment score − posttreatment score)/SE_dif_. After that, patients were classified into four categories based on their RCI, combining statistical and clinical significance^35^: *deterioration* (negative change ≤ −1.96 SE_dif_), *no change* (>−1.96 and <1.96 SE_dif_), *improvement* (positive change ≥ 1.96), or *recovery* (positive change and clinical cut‐off^17^ [FSI severity composite score ≤ 3]). We tested for differences between the intervention and control group by carrying out chi‐square (X^2^) tests.

### 3.8 Sample size

Sample size calculations^16^ showed that we needed 164 participants with complete 12‐week measures in the intervention and control group (total N = 328) to detect an improvement in the primary outcome with a minimal effect size of η^2^ = 0.10 (α = .05, 1‐β = 0.95), using the simplest between/within‐group comparison (*F* tests ‐ RM‐ANOVA with within‐between interaction) in G*Power 3.1.

## 4 RESULTS

### 4.1 Study sample

**FIGURE 1:**
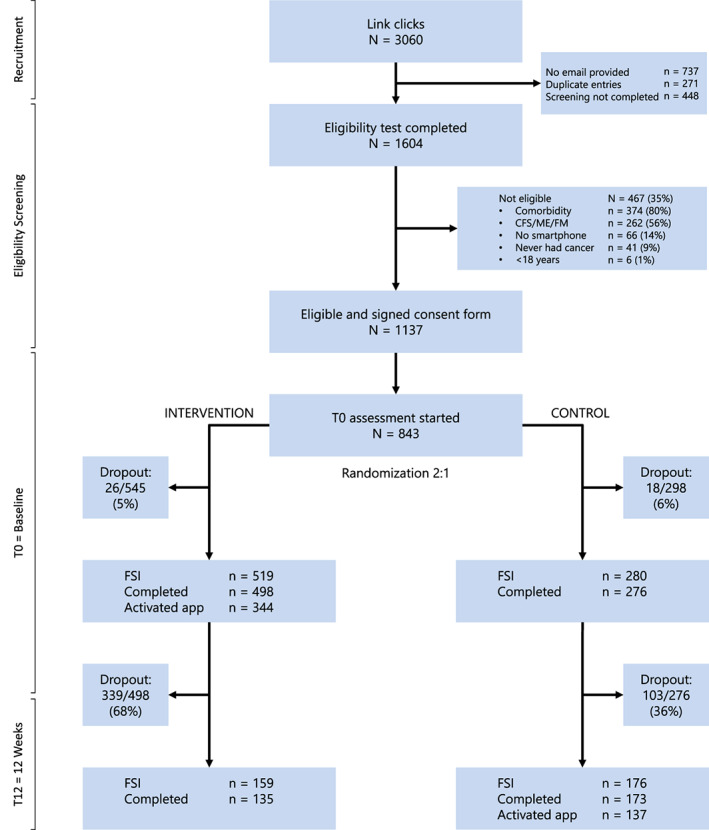
Participant flow (CONSORT diagram) in the Untire app study. The figure presents the FSI at T0 and at T12, the number of completed assessments, and the number of participants who underwent the intervention (i.e., activated the app)

From the 3.060 persons who showed interest in the Untire app study, 1137 met the study criteria and gave informed consent, and 799 participants completed the primary outcome at baseline (Figure 1). As shown in Table 1, the vast majority of the sample was female (92%), middle‐aged (55.5 ± 9.8 years), and moderate to severely fatigued (severity: 6.5 ± 1.4; interference: 5.8 ± 2.0). Most participants reported being a cancer survivor. The baseline characteristics of T12‐completers and T12‐dropouts differed concerning the percentage of participants who received help to manage their fatigue before (T12‐completers: 13%, n = 44; T12‐noncompleters: 8%, n = 35). The intervention participants who activated the app had a slightly higher level of education than those who did not activate the app (*P* < .02).

**TABLE 1:** Baseline characteristics of study participants who completed at least the baseline fatigue assessment in the intervention or control group

	All patients	Intervention	Control
	(N = 799)	(n = 519)	(n = 280)
Demographics			
Female, N (%)	730 (91.8)	477 (92.4)	253 (90.7)
Age, M ± SD	55.5 ± 9.79	56.7 ± 9.99	56.2 ± 9.42
Country, N (%)			
Australia	54 (6.8)	36 (6.9)	18 (6.4)
Canada	53 (6.6)	36 (6.9)	17 (6.1)
United Kingdom	575 (72.0)	370 (71.3)	205 (73.2)
United States	106 (13.3)	70 (13.5)	36 (12.9)
Other^a^	11 (1.4)	7 (1.3)	4 (1.4)
Education, N (%)^b^			
Low	151 (18.9)	92 (17.7)	59 (21.1)
Moderate	306 (38.3)	202 (38.9)	104 (37.1)
High	342 (42.8)	225 (43.2)	117 (41.8)
Health characteristics			
Cancer type, N (%)^c^			
Head/neck	24 (3.0)	14(2.7)	10 (36)
Digestive organs	66 (8.3)	40 (7.7)	26 (9.3)
Respiratory	27 (3.4)	20 (3.9)	7 (2.5)
Skin	17 (2.1)	14 (2.7)	3 (1.1)
Bone	23 (2.9)	13 (2.5)	10 (3.6)
Breast	490 (61.3)	308 (59.3)	182 (65.0)
Female sexual organs	73 (9.1)	54 (10.4)	19 (6.8)
Male sexual organs	15 (1.9)	10 (1.9)	5 (1.8)
Urinary tract	23 (2.9)	18 (3.5)	5 (1.8)
Hematology	70 (8.8)	48 (9.2)	22 (7.9)
Endocrine glands	5 (2.3)	13 (2.5)	18 (1.8)
Eye	2 (0.3)	2 (0.4)	0 (0.0)
Central Nervous System	8 (10)	6 (1.2)	2 (0.7)
Other	22 (2.8)	16 (3.1)	6 (2.1)
Cancer treatment, N (%)^c^			
Surgery	602 (75.3)	389 (75.0)	213 (76.1)
Radiation Therapy	484 (60.6)	309 (59.5)	175 (62.5)
Chemotherapy	573 (71.7)	369 (71.1)	204 (72.9)
Immunotherapy	56 (7%)	45 (8.7%)	11 (3.9)
Stem cell transplant	17 (2.1)	11 (2.1)	6 (2.1)
Hormone therapy	304 (38.0)	187 (36.0)	117 (41.8)
Other treatment(s)	107 (13.4)	66 (12.7)	41 (14.6)
No treatment	12 (1.5)	8 (1.5)	4 (1.4)
Cancer status (survivors), N (%)	485 (60.7)	313 (60.3)	172 (61.4)
Comorbidities N (%)^d^	332 (41.6)	211 (40.7)	121 (43.2)
Fatigue			
Considered themselves fatigued, N (%)	782 (97.9)	511 (98.5)	271 (96.8)
Received help for fatigue, N (%)	79 (10.1)	47 (9.2)	32 (11.8)
Fatigue Symptom Inventory (FSI)			
Severity, *M* ± SD	6.5 ± 1.4	6.5 ± 1.4	6.6 ± 1.4
Interference, *M* ± SD	5.8 ± 2.0	5.7 ± 1.9	5.8 ± 2.0
Quality of life			
Past week (EORTC‐QLQ‐30), *M* ± SD	3.9 ± 1.2	4.0 ± 1.2	3.9 ± 1.2
Overall on average, *M* ± SD	4.3 ± 1.2	4.3 ± 1.2	4.4 ± 1.2
Motivation^e^			
To manage fatigue, *M* ± SD	–	8.0 ± 2.1	–
To work with the app, *M* ± SD	–	8.2 ± 1.9	–

*Note*: This sample includes participants who completed at least the FSI at baseline (thus the sample used for the ITT analyses).

^a^ Countries: Other = the Netherlands (n = 4), Denmark (n = 1), Greece (n = 1), Oman (n = 1), Philippines (n = 1), Ireland (n = 1), South Africa (n = 1), United Arab Emirates (n = 1).

^b^ Education: Lower educated = Primary school and High school; Middle = Associative degree or apprenticeship; High = University (Bachelor, Master, or higher).

^c^ Cancer type and cancer treatment: Participants could have more than one cancer type and could have received more than one cancer treatment, therefore the respective percentages do not sum up to 100%.

^d^ Comorbidities: Diagnosed with any other disease(s) or nonsevere disorder(s) besides cancer.

^e^ Motivation: 11‐point Likert scale from 0 (not motivated) to 10 (as motivated as I could be); only assessed in the intervention group (n = 498).

### 4.2 Does fatigue improve significantly after 12‐weeks of app access?

**FIGURE 2:**
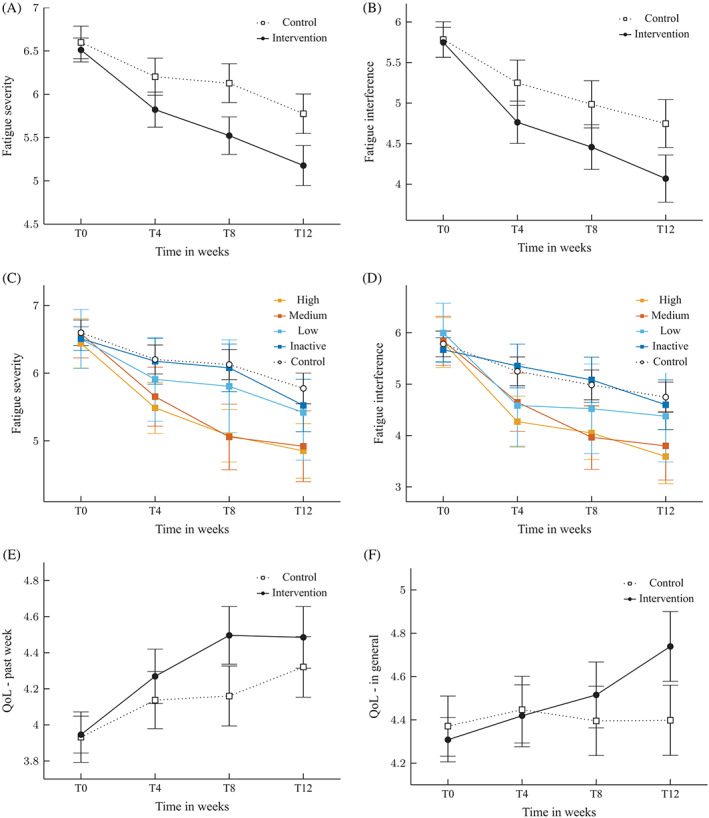
Change in levels of fatigue severity, A and in fatigue interference, B, over time and between conditions. Sensitivity analyses for fatigue severity, C and fatigue interference, D, based on four groups of app use (high, medium, low, nonusers' participants) and waiting‐list control participants. Change in overall QoL during the past week, E, and change in overall QoL on average over time and between conditions, F. Error bars present the 95% confidence interval. QoL, quality of life

After 12‐weeks, participants in the intervention group showed significantly reduced levels of fatigue severity and interference as compared with participants in the control group (*F*(3,1912) = 4.55, *P* < .01, *d* = 0.40, and *F*(3,1912) = 4.10, *P* < .01, *d* = 0.35), see Table 2 and Figure 2. These results were confirmed by the T12‐completers analysis (Appendix S1, Supporting Information 1). Concerning the degree of app use, “high users” used the app ≥9 days, “medium users” used the app ≥3 days, “low users” used the app ≥1 day. The reduction in fatigue severity and interference was seen, especially in participants who showed high and medium app use, in comparison to nonusers and participants in the control group (*P*'s < .02; see Figure 2, and Supporting Information 2). The levels of fatigue in low app users, nonusers, and control participants did not statistically differ after 12‐weeks (*P*'s > .05).

**TABLE 2:** Generalized linear mixed model (GLMM) results for the main effects of group and time, and the interaction effect of group by time (ITT) on fatigue severity, fatigue interference, and quality of life of past week and overall quality of life on average

Outcome	Intervention		Control		Group		Time		Group*Time			
Fatigue severity	N	M ± SD	N	M ± SD	*F*	*P*	*F*	*P*	*F*	95% CI	*P*	*d*
T0	519	6.51 ± 1.37	280	6.60 ± 1.43								
T4	213	5.81 ± 1.74	204	6.21 ± 1.57								
T8	185	5.48 ± 2.00	184	6.12 ± 1.56								
T12	159	5.11 ± 2.09	176	5.77 ± 1.79	16.21	<.01	59.45	<.01	−4.55	−0.85 to −1.73	<.01	0.40
Fatigue interference												
T0	519	5.75 ± 1.92	280	5.78 ± 2.04								
T4	213	4.72 ± 2.25	204	5.25 ± 2.11								
T8	185	4.36 ± 2.44	184	5.01 ± 2.20								
T12	159	3.98 ± 2.43	176	4.77 ± 2.35	9.12	<.01	68.82	<.01	−4.10	−1.05 to −0.235	<.01	0.35
Quality of life—Past week												
T0	516	3.95 ± 1.15	278	3.93 ± 1.17								
T4	211	4.29 ± 1.28	204	4.14 ± 1.19								
T8	182	4.54 ± 1.21	182	4.18 ± 1.18								
T12	157	4.57 ± 1.28	175	4.35 ± 1.20	4.37	.04	23.47	<.01	−2.34	−0.40 to 0.75	.07	0.15
Quality of life—On average												
T0	516	4.31 ± 1.18	278	4.37 ± 1.18								
T4	211	4.44 ± 1.27	204	4.45 ± 1.14								
T8	182	4.57 ± 1.19	182	4.40 ± 1.13								
T12	157	4.80 ± 1.16	175	4.42 ± 1.15	1.36	.24	5.46	<.01	−4.67	0.18 to 0.63	<.01	0.32

*Note*: This sample includes participants who completed at least the FSI at baseline (ITT).

Fatigue Severity = Fatigue Symptom Inventory (FSI ‐ Severity composite score ((items 1+2+3)/3)); Fatigue Interference = Fatigue Symptom Inventory (FSI – Interference composite score ((items 5+6+7+8+9+10+11)/7); Quality of life ‐ Past week: EORTC‐QLQ‐30 = Quality of Life questionnaire ‐ past week; *d* = Cohens *d* effect size (0.02 ~ small, 0.5 ~ medium, 0.8 ~ large). Data are given as mean ± SD.

The clinical relevance analysis for fatigue interference showed differences in the number of people who recovered, improved, not‐changed, and deteriorated, favoring the intervention group (*P* = .04), whereas for fatigue severity, no statistically significant difference was found (*P* = .05), see Supporting Information 3. With regard to both fatigue severity and interference, significantly more people recovered in the intervention group vs the control group after 12‐weeks (*P*'s = .02).

### 4.3 Does QoL improve significantly after 12‐weeks of app access?

QoL during the past week (EORTC‐QLQ‐30) did not differ significantly between conditions after 12‐weeks (*F*(3,1897) = 2.34, *P* = .07, *d* = 0.15), whereas levels of QoL on average did improve significantly in the intervention group from T0 to T12 as compared with the control group (*F*(1,334) = 4.67, *P* < .01, *d* = 0.32), see Figure 2 and Supporting Information 1.

### 4.4 Moderation of intervention effects

The variables age, education, and cancer status were taken as moderators into account. The significant intervention effect on fatigue interference was moderated by age (*F*(3,1887) = 2.67, *P* = .046), with slightly stronger improvements in younger intervention participants (≤56 vs. >56). Education and cancer status did not moderate any of the intervention effects (all *P*'s > .05; see Supporting Information 4 and 5).

## 5 DISCUSSION

This international waiting‐list RCT examined whether the number one side effect of cancer and cancer treatment (ie, CRF) can be effectively reduced with a self‐management app for cancer patients and survivors. While just a few web‐based/eHealth interventions targeted at reducing fatigue in cancer patients and survivors exist,^5^, ^6^, ^7^, ^8^, ^9^, ^10^ the effects of mHealth delivery on fatigue remain unclear.^3^ Our data showed that having 12‐week access to the Untire app significantly improved levels of fatigue and QoL on average. These effects were found, especially among those participants who used the app to a medium or high degree, which was in practice related to ≥3 days of app use. In addition, significantly more participants in the intervention group showed full recovery from fatigue when compared with the control group. Although we only explored moderators (ie, age, level of education, and cancer status) and more research on potential moderating factors is needed, the present results suggest that the self‐management app could be beneficial for different types of individuals.

### 5.1 Clinical implications

Our study demonstrated small‐to‐moderate treatment effects (*d* = 0.35‐0.40) of the Untire intervention, which is larger than other self‐management eHealth interventions (ie, *d* = 0.21^10^) but smaller than the effects of therapist‐guided online interventions (ie, *d* = 0.71^3^, ^36^) and face‐to‐face therapy (ie, *d* = 0.45^36^; or *d* = 0.74^24^) targeted at CRF. However, it is important to note that with self‐management (ie, not therapist guided) mHealth interventions, a higher number of patients and survivors can be reached and helped simultaneously at much lower costs. Although the high attrition rates in the intervention group reflect that a self‐management mHealth intervention might not be suitable for everyone, we assume that an app due to its low‐threshold supply could be especially helpful for patients who would otherwise not engage in any form of treatment. In line with this, we appeared to have attracted participants who did not receive any help previously.

### 5.2 Study limitations

In addition to our study's international focus and large‐scale set‐up, we addressed the issue of widespread CRF by an innovative low‐threshold mHealth intervention. A specific advantage of the Untire app itself is its flexibility, as the patients are in the lead to select the content or exercises that match their interests and needs instead of following a predetermined week‐by‐week program. Current in‐app engagement strategies include different modes of content delivery, persuasive elements, reminders, and social support. Increasing personalized feedback may further engage participants and foster retention.

Carrying out a waiting‐list RCT helped us to demonstrate causality, and to control for potential spontaneous improvements during the study period. A drawback of this design is that it might artificially inflate intervention effect estimates since participants in the control group could have been influenced by design to literally “wait‐to‐change” and thus do not improve.^37^ However, this does not seem to be the case in our study, since control participants also showed improvements. Another limitation is that, in order to limit patient burden, QoL was assessed with two questions instead of using a complete, validated QoL questionnaire.

A drawback of using an online recruitment procedure is that multiple registrations by one person are possible. We removed duplicate registrations based on email address and/or name. In the study sample, the percentage of persons who completed the registration more than once did not differ between conditions, assuming that those attempts were not motivated by the idea of receiving immediate app access while being initially allocated to the waitinglist‐control group (ie, no evidence for a broken randomization). Another drawback of online recruitment is the lack of access to hospital records, meaning that we cannot validate medical information (eg, cancer type and status). However, participants had nothing to gain by providing invalid medical information. Another limitation is that remotely conducted mHealth studies regularly show high attrition rates concerning study assessments and intervention participation (in our case, app use). High dropout rates and decreasing app use may be common,^28^, ^30^, ^38^ as the choice to quit is uncomplicated owing to the impersonal character of the online study and intervention.^39^ We intended to counteract the *law of attrition* ^39^ by sending reminder e‐mails supporting participants to complete study assessments. Due to the design and the methodology used, it was not possible to have personal contact with the participants, and increasing adherence by sending reminders was partially successful. Low retention rates prohibit any inferences about participants who dropped out and might introduce selection bias. It is important to realize that unequal dropout rates in intervention and control groups do not necessarily imply that effect estimates are biased.^40^ As aforementioned, our data analysis did not find any substantial differences when comparing baseline characteristics (Table 1) of T12‐completers with T12‐dropouts, and the results were consistent between analytic strategies (ie, ITT and T12‐completers). Furthermore, we acknowledge that blind analyses would have been the optimal way to eliminate the experimenter's bias. We aimed to reduce experimenter bias by publishing the methodology and the statistical analysis plan in the Trial Registry prior to analyzing our data.^16^

We should be aware that the recruitment strategy may have influenced our study sample, and that our findings might be limited to middle‐aged female breast cancer patients who are using Facebook. However, our sample is likely to represent future users who might find the Untire app in a similar way, that is, through finding information on the internet or Facebook. This idea is supported by data from users who downloaded and used the Untire app after the study had been completed, which showed that most users were indeed middle‐aged and female.

### 5.3 Future research

Future research could explore the mechanisms behind the app (ie, possible mediators of the treatment effect, such as fatigue catastrophizing, mindfulness, physical activity, or sleep) to understand the underlying processes determining the reduction of fatigue. To further validate the study results, also participants in non‐English speaking countries should be targeted. Besides online recruitment, also other pathways of introducing the app to the end‐user (ie, introduction to the patient by a healthcare professional) could be explored concerning differences in sample characteristics, dropout, adherence, and effectiveness.

## 6 CONCLUSIONS

This study shows that a self‐management app can support cancer patients and survivors in managing their fatigue and QoL, and suggests that besides existing face‐to‐face therapy or therapist‐guided online interventions, also a low‐threshold mHealth app can be an effective treatment solution. The Untire app could present a scalable opportunity in supporting people worldwide experiencing disabling fatigue.

## CONFLICT OF INTEREST

The authors declare that they have no conflict of interest. The University Medical Center Groningen received funding from Tired of Cancer BV., the developer of the Untire app, to study its effectiveness independently. Independence is declared in a research agreement.

## ETHICAL APPROVAL STATEMENT

The Untire app study has been approved by the Medical Ethical Committee of the University Medical Center Groningen (UMCG), the Netherlands. Hereafter, the study received either ethical approval or a waiver from authorized institutions in the four English‐speaking countries targeted (ie, Australia, Canada, the United Kingdom, and the United States). The trial has been registered on November 29, 2019 on the Netherlands Trial Registry (NL6642).

## Supporting information


**Appendix S1**: Supporting Information

## Data Availability

The data that support the findings of this study are available from the corresponding author upon reasonable request.

## Appendix A.

Untire app modules and description.

**Table:**

Untire app components	
Daily program	Description
My themes	Receive psycho‐education and exercises on topics such as fear, worry, anxiety, and sleep.
Basics	Get basic education about different sources of fatigue (ie, the other themes) and receive an overview of how to work with the Untire app, including the rationale of the *vase of energy*.
Fatigue	Receive psycho‐education and medical background information and exercises about CRF.
Anxiety	Receive information on the physiology and the harmlessness of anxiety, as well as several tips on how to cope with fear.
Worry	Learn to distinguish between thoughts and facts, and learn that thoughts are malleable. Furthermore, participants receive challenging questions to explore their thoughts.
Boundaries	Learn to accept that energy levels are often lower as compared with before your disease. Furthermore, learn to understand that time‐outs may be needed and that you can actively manage your boundaries. Gain insight into your boundaries in order to prevent crossing them repeatedly.
Sleep	Learn about different factors related to sleep problems and the vicious circle of insomnia, which may influence (quality of) sleep. Receive suggestions on how to improve your sleeping behavior.
Self‐care	Improve your self‐compassion by receiving 20 suggestions for self‐care.
Nutrition	Receive general information on healthy nutrition based on the latest scientific research.
My exercises	Engage in 17 different MBSR exercises to identify, manage, and release stress (ie, “attention to the breath” or “body scan”).
My physical activity	Receive information and exercises on three different aspects of the Untire physical exercise training program to increase levels of physical activity and muscle strength.
Planning	Receive several exercises and tips to deal with limited levels of energy, such as agenda taking and indicate how the upcoming days will be about activities and energy levels.
Build up energy	Build‐up your energy by carrying out daily physical activity based on guidance into 30 minutes a day (daily increasing difficulty). Receive guidance in improving sedentary behaviors through information, exercises, and tips.
Power	Receive guidance to build‐up power and muscle strength through information, exercises, and tips.
Tip of the day	Tips are ideas or quotes based on positive psychology, which should help to get into a good mood (for instance, writing a gratitude letter).
Assessments	
Quick scan	Receive weekly evaluations involving four questions about wellbeing as well as three questions about energy (part of the *vase of energy*). Via the personal profile, you can see your progress anytime.
Fatigue	How was your fatigue this last week?
Burden	How was your burden this last week?
Happiness	How happy have you been this last week?
Satisfaction	How satisfied have you been this last week?
Vase of energy	Receive three questions presented visually by the vase of energy: What gives you energy? What costs you energy? What is your energy leak? The weekly pattern provides insights into energy levels.
