# Supplementary material for: Beating cancer‐related fatigue with the Untire mobile app: Results from a waiting‐list randomized controlled trial
Source: Psychooncology. 2020 Oct 11;29(11):1823–34. doi: 10.1002/pon.5492 (PMC7756868; doi:10.1002/pon.5492)
Supplement: Supplementary file 1 — Appendix S1: Supporting Information [file PON-29-1823-s001.docx]

**Supporting Information**

**Supporting information 1**

*Generalized linear mixed model (GLMM) results for the main effects of group and time, and the interaction effect group by time (T12-completers) on fatigue severity, fatigue interference, and quality of life of past week and overall quality of life on average.*

|  |  | Intervention | | |  |  | Control | |  |  | Group | |  | Time | |  | Group*Time | | | |
| --- | --- | --- | --- | --- | --- | --- | --- | --- | --- | --- | --- | --- | --- | --- | --- | --- | --- | --- | --- | --- |
| Outcome |  | N |  | M ± SD | ___ |  | N | M ± SD | ___ |  | *F* | *P* | ___ | *F* | *P* | *___* | *F* | 95% CI | *P* | *d* |
| Fatigue Severity | | | | | | | | | | | | | | | | | | | | |
| T0 |  | 159 |  | 6.47 ± 1.42 |  |  | 176 | 6.60 ± 1.39 |  |  |  |  |  |  |  |  |  |  |  |  |
| T4 |  | 136 |  | 5.65 ± 1.87 |  |  | 161 | 6.21 ± 1.50 |  |  |  |  |  |  |  |  |  |  |  |  |
| T8 |  | 134 |  | 5.41 ± 2.00 |  |  | 155 | 6.13 ± 1.49 |  |  |  |  |  |  |  |  |  |  |  |  |
| T12 |  | 159 |  | 5.11 ± 2.09 |  |  | 176 | 5.77 ± 1.79 |  |  | 12.55 | .000 |  | 43.90 | .000 |  | -4.55 | _-.92 to -1.59_ | **.013** | .389 |
| Fatigue Interference | | | | | | | | | | | | | | | | | | | | |
| T0 |  | 159 |  | 5.69 ± 1.88 |  |  | 176 | 5.85 ± 2.00 |  |  |  |  |  |  |  |  |  |  |  |  |
| T4 |  | 136 |  | 4.53 ± 2.30 |  |  | 161 | 5.30 ± 2.11 |  |  |  |  |  |  |  |  |  |  |  |  |
| T8 |  | 134 |  | 4.30 ± 2.45 |  |  | 155 | 5.00 ± 2.16 |  |  |  |  |  |  |  |  |  |  |  |  |
| T12 |  | 159 |  | 3.98 ± 2.43 |  |  | 176 | 4.77 ± 2.35 |  |  | 8.20 | .004 |  | 53.19 | .000 |  | -2.95 | _-1.08 to -.17_ | **.032** | .355 |
| Quality of life – Past week | | | | | | | | | | | | | | | | | | | | |
| T0 |  | 159 |  | 4.03 ± 1.24 |  |  | 176 | 3.97 ± 1.07 |  |  |  |  |  |  |  |  |  |  |  |  |
| T4 |  | 136 |  | 4.51 ± 1.23 |  |  | 161 | 4.17 ± 1.18 |  |  |  |  |  |  |  |  |  |  |  |  |
| T8 |  | 133 |  | 4.64 ± 1.27 |  |  | 155 | 4.17 ± 1.15 |  |  |  |  |  |  |  |  |  |  |  |  |
| T12 |  | 157 |  | 4.57 ± 1.28 |  |  | 175 | 4.35 ± 1.20 |  |  | 7.00 | .008 |  | 17.13 | .000 |  | -2.43 | -.10 to .453_ | .064 | .197 |
| Quality of life – On Average | | | | | | | | | | | | | | | | | | | | |
| T0 |  | 159 |  | 4.30 ± 1.21 |  |  | 176 | 4.39 ± 1.16 |  |  |  |  |  |  |  |  |  |  |  |  |
| T4 |  | 136 |  | 4.59 ± 1.26 |  |  | 161 | 4.45 ± 1.14 |  |  |  |  |  |  |  |  |  |  |  |  |
| T8 |  | 133 |  | 4.65 ± 1.19 |  |  | 155 | 4.40 ± 1.05 |  |  |  |  |  |  |  |  |  |  |  |  |
| T12 |  | 157 |  | 4.80 ± 1.16 |  |  | 175 | 4.42 ± 1.15 |  |  | 2.28 | .131 |  | 6.30 | .000 |  | -5.43 | _-.64 to -.14_ | **.001** | .334 |

*Note.* This sample includes participants who completed at least the FSI at baseline and the FSI 12-week outcome assessment (T12-completers). Fatigue Severity = Fatigue Symptom Inventory (FSI - Severity composite score ((items 1+2+3)/3)); Fatigue Interference = Fatigue Symptom Inventory (FSI - Severity composite score ((items 5+6+7+8+9+10+11)/7); EORTC-QLQ-30 = Quality of Life questionnaire (i.e., Quality of life – On Average); *d* = Cohens d (.02 ~ small, .5 ~ medium, .8 ~ large). Data are given as mean ± standard deviation.

**Supporting information 2**

*Sensitivity analysis for fatigue severity and interference, comparing outcomes of the 33% most active app users ‘high users’, the middle 33% ‘medium’ users, the least active 33% ‘low’ users, nonusers, and control participants.*

| Group vs. reference group | Fatigue | | | | | | | |
| --- | --- | --- | --- | --- | --- | --- | --- | --- |
|  | Severity | | |  |  | Interference | | |
|  | Contrast estimate | 95% CI | *p* |  |  | Contrast estimate | 95% CI | *p* |
| High vs. medium | -.09 | -.51 to .33 | .67 |  |  | -.13 | -.72 to .45 | .66 |
| High vs. low | -.45 | -.95 to .06 | .08 |  |  | -.44 | -1.13 to .25 | .21 |
| High vs. nonusers | -.61 | -.95 to -.26 | **<.01** |  |  | -.75 | -1.23 to -.27 | **<.01** |
| High vs. control | -.71 | -1.03 to -.39 | **<.01** |  |  | -.76 | -1.21 to -.31 | **<.01** |
| Medium vs. low | -.36 | -.87 to .16 | .18 |  |  | -.31 | -1.01 to .40 | .41 |
| Medium vs. nonusers | -.52 | -.89 to -.15 | **<.01** |  |  | -.61 | -1.12 to -.11 | **.02** |
| Medium vs. control | -.62 | -.97 to -.28 | **<.01** |  |  | -.63 | -1.11 to -.15 | **.01** |
| Low vs. nonusers | -.16 | -.62 to .30 | .50 |  |  | -.31 | -.93 to .32 | .34 |
| Low vs. control | -.27 | -.71 to .18 | .24 |  |  | -.32 | -.92 to .28 | .30 |
| Nonusers vs. control | -.11 | -.36 to .15 | .42 |  |  | -.02 | -.36 to .33 | .93 |
| Time*Group |  | *F*(12,1900)=2.57 | **.02** |  |  |  | *F*(12,1900)=3.53 | **<.01** |

*Note.* Groups: ‘High users’ used the app ≥9 days, ‘medium users,’ used the app ≥3 days, ‘low users,’ used the app ≥1 day, ‘nonusers’ did not download and activate the app, and participants in the control group (did not receive access to the Untire app).

**Supporting information 3**

*Clinical relevance analysis for T12-completers in intervention vs. control group.*

| Measure | Recovered | |  | Improved | |  | No change | |  | Deteriorated | |  | Test of difference | | |
| --- | --- | --- | --- | --- | --- | --- | --- | --- | --- | --- | --- | --- | --- | --- | --- |
|  | Intervention | Control |  | Intervention | Control |  | Intervention | Control |  | Intervention | Control |  | *χ^2^* | *P* | *V* |
| Fatigue Severity | | |  |  |  |  |  |  |  |  |  |  |  |  |  |
|  | 22 (14%) | 11 (6%) |  | 43 (27%) | 39 (22%) |  | 83 (52%) | 110 (63%) |  | 11 (7%) | 16 (9%) |  | 7.72 | .05 | .15 |
| Fatigue Interference | | |  |  |  |  |  |  |  |  |  |  |  |  |  |
|  | 48 (30%) | 33 (19%) |  | 34 (19%) | 32 (20%) |  | 63 (40%) | 95 (54%) |  | 17 (10%) | 15 (8%) |  | 8.61 | **.04** | .16 |

*Note.* Fatigue Severity = Fatigue Symptom Inventory (FSI - Severity composite score ((items 1+2+3)/3)); Fatigue Interference = Fatigue Symptom Inventory (FSI - Severity composite score ((items 5+6+7+8+9+10+11)/7); Change: *Recovered* [Positive change (improvement), statistically reliable (RCI ≤ -1.96), and moves from above clinical cut-off to below cut-off]; *Improved* [Positive change (improvement), and statistically reliable (RCI ≤ -1.96)]; *No change* [No change, not statistically reliable ( -1.96 ≤ RCI ≤ 1.96)]; *Deteriorated* [Negative change (deterioration), and statistically reliable (RCI ≥ 1.96)]. Effect size: Cramer’s V (*V*). The population standard deviation ‘S’ is estimated from the total sample population (N=799, SD_FSI_severity = 1.39, SD_FSI_interference = 1.96

**Supporting information 4**

*Generalized linear mixed model (GLMM) results for the corrected model, main effects of group, time, and moderator variable, all two-way interactios, and the three-way interaction effect on fatigue severity, fatigue interference, QoL of the past week, and overall QoL on average.*

|  |  | Fatigue Severity | | | |  | Fatigue Interference | | | |  | QoL past week | | | |  | QoL on average | | | |
| --- | --- | --- | --- | --- | --- | --- | --- | --- | --- | --- | --- | --- | --- | --- | --- | --- | --- | --- | --- | --- |
|  |  | F | df1 | df1 | p |  | F | df1 | df2 | p |  | F | df1 | df2 | p |  | F | df1 | df2 | p |
| Age | |  |  |  |  |  |  |  |  |  |  |  |  |  |  |  |  |  |  |  |
|  | Corrected Model | 15.00 | 15 | 1887 | .000 |  | 16.56 | 15 | 1887 | .000 |  | 6.85 | 15 | 1872 | .000 |  | 2.56 | 15 | 1872 | .001 |
|  | time | 62.16 | 3 | 1887 | .000 |  | 70.50 | 3 | 1887 | .000 |  | 25.03 | 3 | 1872 | .000 |  | 6.03 | 3 | 1872 | .000 |
|  | condition | 18.71 | 1 | 1887 | .000 |  | 9.78 | 1 | 1887 | .002 |  | 4.97 | 1 | 1872 | .026 |  | 1.80 | 1 | 1872 | .180 |
|  | age | .16 | 1 | 1887 | .688 |  | .52 | 1 | 1887 | .473 |  | 2.07 | 1 | 1872 | .150 |  | 3.79 | 1 | 1872 | .052 |
|  | time * age group | 3.94 | 3 | 1887 | .008 |  | 2.78 | 3 | 1887 | .040 |  | 4.05 | 3 | 1872 | .007 |  | .54 | 3 | 1872 | .656 |
|  | condition * age group | 2.46 | 1 | 1887 | .117 |  | .29 | 1 | 1887 | .588 |  | .15 | 1 | 1872 | .701 |  | .62 | 1 | 1872 | .433 |
|  | condition * time | 5.59 | 3 | 1887 | **.001** |  | 4.77 | 3 | 1887 | **.003** |  | 2.48 | 3 | 1872 | .060 |  | 4.90 | 3 | 1872 | **.002** |
|  | time * condition * age group | 1.96 | 3 | 1887 | .118 |  | 2.67 | 3 | 1887 | **.046** |  | 1.84 | 3 | 1872 | .138 |  | .70 | 3 | 1872 | .554 |
| Education | |  |  |  |  |  |  |  |  |  |  |  |  |  |  |  |  |  |  |  |
|  | Corrected Model | 10.32 | 23 | 1896 | .000 |  | 10.89 | 23 | 1896 | .000 |  | 5.10 | 23 | 1881 | .000 |  | 2.80 | 23 | 1881 | .000 |
|  | time | 52.04 | 3 | 1896 | .000 |  | 55.59 | 3 | 1896 | .000 |  | 17.07 | 3 | 1881 | .000 |  | 3.40 | 3 | 1881 | .017 |
|  | condition | 16.09 | 1 | 1896 | .000 |  | 8.07 | 1 | 1896 | .005 |  | 1.26 | 1 | 1881 | .262 |  | .35 | 1 | 1881 | .554 |
|  | education | 9.57 | 2 | 1896 | .000 |  | 7.08 | 2 | 1896 | .001 |  | 9.94 | 2 | 1881 | .000 |  | 12.36 | 2 | 1881 | .000 |
|  | time * education | .19 | 6 | 1896 | .979 |  | 1.13 | 6 | 1896 | .345 |  | 1.15 | 6 | 1881 | .334 |  | .60 | 6 | 1881 | .734 |
|  | condition * education | 1.82 | 2 | 1896 | .162 |  | .28 | 2 | 1896 | .759 |  | 2.46 | 2 | 1881 | .086 |  | 1.33 | 2 | 1881 | .265 |
|  | condition * time | 4.81 | 3 | 1896 | **.002** |  | 2.63 | 3 | 1896 | **.048** |  | 2.53 | 3 | 1881 | .056 |  | 3.14 | 3 | 1881 | **.024** |
|  | time * condition * education | 1.55 | 6 | 1896 | .157 |  | .92 | 6 | 1896 | .477 |  | .46 | 6 | 1881 | .841 |  | 1.14 | 6 | 1881 | .335 |
| Cancer status | |  |  |  |  |  |  |  |  |  |  |  |  |  |  |  |  |  |  |  |
|  | Corrected Model | 14.12 | 15 | 1904 | .000 |  | 15.68 | 15 | 1904 | .000 |  | 6.27 | 15 | 1889 | .000 |  | 2.67 | 15 | 1889 | .001 |
|  | time | 52.69 | 3 | 1904 | .000 |  | 60.78 | 3 | 1904 | .000 |  | 22.51 | 3 | 1889 | .000 |  | 5.15 | 3 | 1889 | .002 |
|  | condition | 11.67 | 1 | 1904 | .001 |  | 8.17 | 1 | 1904 | .004 |  | 4.86 | 1 | 1889 | .028 |  | 1.94 | 1 | 1889 | .164 |
|  | cancer status | 1.35 | 1 | 1904 | .246 |  | 4.86 | 1 | 1904 | .028 |  | 8.48 | 1 | 1889 | .004 |  | 3.12 | 1 | 1889 | .078 |
|  | time * cancer status | 1.52 | 3 | 1904 | .208 |  | .63 | 3 | 1904 | .594 |  | .33 | 3 | 1889 | .803 |  | 1.62 | 3 | 1889 | .183 |
|  | condition * cancer status | 3.42 | 1 | 1904 | .065 |  | .01 | 1 | 1904 | .906 |  | .41 | 1 | 1889 | .524 |  | 1.31 | 1 | 1889 | .253 |
|  | condition * time | 3.89 | 3 | 1904 | **.009** |  | 3.43 | 3 | 1904 | **.016** |  | 2.16 | 3 | 1889 | .091 |  | 4.60 | 3 | 1889 | **.003** |
|  | time * condition * cancer status | .12 | 3 | 1904 | .947 |  | .84 | 3 | 1904 | .473 |  | .05 | 3 | 1889 | .985 |  | .46 | 3 | 1889 | .707 |

Note: Age (mean = 56.49, median = 56.00) younger vs. older split from 56 years to derive at two equal-sized groups.

**Supporting information 5**


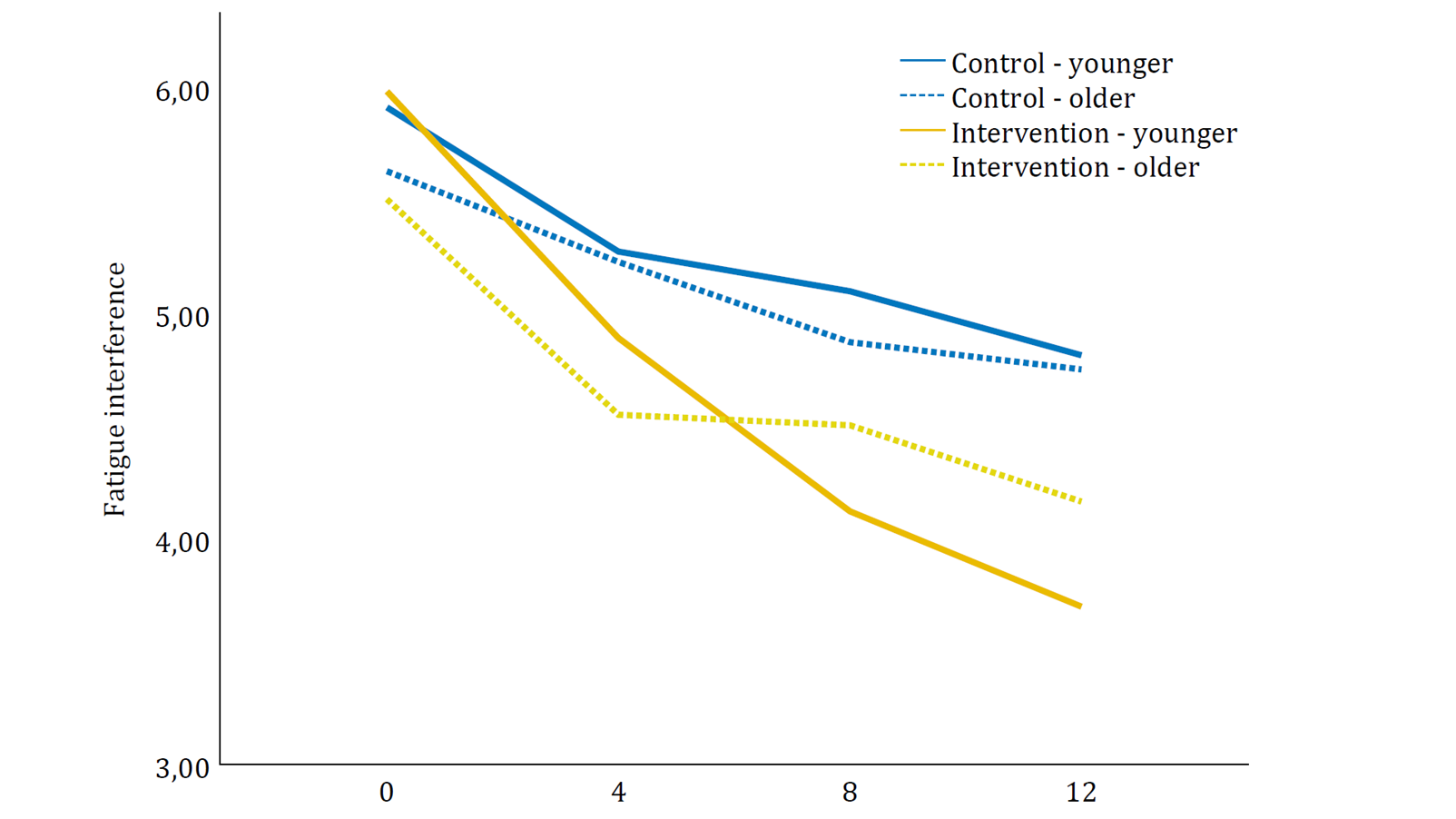


Change in levels fatigue interference over time, between conditions, by moderator age group (i.e., younger vs. older). Cases with missing values are excluded listwise. Age groups were determined by median/mean split of the age variable (i.e. younger ≤ 56 years) .
